# Aberrant DNA Methylation: Implications in Racial Health Disparity

**DOI:** 10.1371/journal.pone.0153125

**Published:** 2016-04-25

**Authors:** Xuefeng Wang, Ping Ji, Yuanhao Zhang, Joseph F. LaComb, Xinyu Tian, Ellen Li, Jennie L. Williams

**Affiliations:** 1 Department of Family, Population and Preventive Medicine, Stony Brook University, Stony Brook, NY, 11794, United States of America; 2 Department of Medicine, Stony Brook University, Stony Brook, NY, 11794, United States of America; 3 Department of Biomedical Informatics, Stony Brook University, Stony Brook, NY, 11794, United States of America; 4 Department of Applied Mathematics and Statistics, Stony Brook University, Stony Brook, NY, 11794, United States of America; 5 Division of Cancer Prevention, Stony Brook University, Stony Brook, NY, 11794, United States of America; 6 Division of Gastroenterology, Stony Brook University, Stony Brook, NY, 11794, United States of America; UCSF / VA Medical Center, UNITED STATES

## Abstract

**Background:**

Incidence and mortality rates of colorectal carcinoma (CRC) are higher in African Americans (AAs) than in Caucasian Americans (CAs). Deficient micronutrient intake due to dietary restrictions in racial/ethnic populations can alter genetic and molecular profiles leading to dysregulated methylation patterns and the inheritance of somatic to germline mutations.

**Materials and Methods:**

Total DNA and RNA samples of paired tumor and adjacent normal colon tissues were prepared from AA and CA CRC specimens. Reduced Representation Bisulfite Sequencing (RRBS) and RNA sequencing were employed to evaluate total genome methylation of 5’-regulatory regions and dysregulation of gene expression, respectively. Robust analysis was conducted using a trimming-and-retrieving scheme for RRBS library mapping in conjunction with the BStool toolkit.

**Results:**

DNA from the tumor of AA CRC patients, compared to adjacent normal tissues, contained 1,588 hypermethylated and 100 hypomethylated differentially methylated regions (DMRs). Whereas, 109 hypermethylated and 4 hypomethylated DMRs were observed in DNA from the tumor of CA CRC patients; representing a 14.6-fold and 25-fold change, respectively. Specifically; CHL1, 4 anti-inflammatory genes (i.e., NELL1, GDF1, ARHGEF4, and ITGA4), and 7 miRNAs (of which miR-9-3p and miR-124-3p have been implicated in CRC) were hypermethylated in DNA samples from AA patients with CRC. From the same sample set, RNAseq analysis revealed 108 downregulated genes (including 14 ribosomal proteins) and 34 upregulated genes (including POLR2B and CYP1B1 [targets of miR-124-3p]) in AA patients with CRC versus CA patients.

**Conclusion:**

DNA methylation profile and/or products of its downstream targets could serve as biomarker(s) addressing racial health disparity.

## Introduction

The incidence and mortality rates of colorectal cancer (CRC) in the United States are higher in African Americans (AAs) as compared to all other ethnic/racial groups [1]. One report illustrated a 30–50% higher rate of CRC mortality in AAs post-diagnosis compared to CAs. Moreover, this racial health disparity continues to expand despite increased CRC screening [2–7]. AAs also develop and are diagnosed with CRC at a younger age compared to CAs [8]. Cumulatively, it is hypothesized that 1) epigenetic or molecular differences elicit this prevalent racial disparity, and 2) socio-economic factors are at least partly responsible for these variances. In addition, the initiation and progression of CRC is linked to chronic intestinal inflammation. In North America, the risk of CRC in patients with inflammatory bowel disease is 2 times greater as compared to the general population [9].

It is well documented that factors such as diet and lack of preventive medical care are influential in incidence and early detection of disease. 12% of all CRC cases, regardless of ethnic background or other demographic factors, are attributed to a Western diet/nutrition [10]. Recent epidemiological studies have concluded that the abundance or deficiency of specific dietary micronutrients increases the risk for development and progression of CRC. For example, dietary folate levels regulate nucleotide synthesis and impact DNA methylation, which in turn alters cell proliferation, DNA repair and genomic stability [11]. Disparity in CRC incidence and ethnic genomic variation may have a direct correlation due in part to ethnic dietary patterns. Importantly somatic mutations may progressively become germline mutations [12]. For example, the *p53* tumor suppressor gene, known to be mutated in over 50% of all human cancers [13], has unique polymorphisms within AAs further contributing to racial disparity seen in CRC patients [14].

Aberrant CpG island hypermethylation at the promoter of tumor suppressor transcription factors [15] and hypomethylation of oncogenes [16] are important mechanisms for gene inactivation or activation, respectively. This aberration is influential in accumulating genomic alterations leading to carcinogenesis. While many studies seek to define methylation patterns in CRC across the broad population, little is known about the role epigenetic differences play in racial/ethnic health disparity. For instance, sporadic CRC caused by promoter hypermethylation of the mismatch repair gene MLH1 could result in underlying genetic predisposition for AA in later generations [17]. Such is true in hereditary non-polyposis CRC with germline mutations in mismatch repair genes MLH1, MLH2, MSH6, and PMS2. Resulting microsatellite instabilities (MSIs) disproportionately occur in AAs compared to CAs which contribute to accelerated CRC progression [18]. In addition, dysregulation of microRNAs (miRNAs) are well-documented across many types of cancers and are potential biomarkers for cancer classification and prognosis [19]. The mechanism underlying miRNA dysregulation in cancer is not fully understood; however, recent studies have shown that epigenetic mechanisms play important roles in the regulation of miRNA expression [20]. Importantly, miRNAs can act as either tumor suppressors by inhibiting oncogenic gene expression or, conversely, as oncomirs by inhibiting tumor suppressor gene expression.

Here, we demonstrate that AA CRC specimens have significantly higher levels of hyper- and hypomethylation versus CA CRC specimens. Comparative analysis was conducted to elucidate epigenetic differences that may be drivers of racial disparity seen in incidence of CRC. Association of aberrant methylation to its effects on downstream gene transcription was assessed by RNA sequencing. Here, we report that hypermethylation of the anti-inflammatory transcription factors NELL1, GDF1, ARHGEF4, and ITGA4 and multiple miRNAs including miR-9-3p and miR-124-3p may be factors driving the disparity observed in incidence of CRC between racial and ethnic groups. We also reveal, as determined by RNA sequencing analysis, that two targets of miR-124-3p (Polymerase (RNA) II Polypeptide B (POLR2B) and Cytochrome P450, Family 1, Subfamily B, Polypeptide 1 (CYP1B1)) are upregulated and 14 ribosomal proteins (RPs) are downregulated in tumors of AA CRC patients. This would suggest that functional and genetic studies are necessary to determine which hyper/hypomethylation events are truly relevant to human tumorigenesis and contribute to racial health disparity.

## Materials and Methods

### Ethics statement

This study was approved by the Washington University (WU) School of Medicine-St. Louis and Stony Brook University (SBU) Institutional Review Boards, approval #93677–16. Tissues were obtained from the WU (http://www.siteman.wustl.edu/ContentPage.aspx?id=243) and SBU human biobanks. The samples and clinical metadata were de-identified, assigned independent patient and sample codes prior to release to the researchers, and qualified for a waiver of consent per 45CFR46.116.d.

### Nucleic acid extraction from WU colon cancer tumor and adjacent normal colon tissue samples

Total DNA and total RNA were prepared by and acquired from the Siteman Cancer Center Tissue Procurement Facility at Washington University (WU)-St. Louis for 6 AA and 7 CA colon cancer patients who underwent colon cancer surgery at Barnes Jewish Hospital. The total RNA was extracted from the 13 pairs of snap-frozen tumors and adjacent matching normal colon using TRIzol followed by lithium precipitation (Invitrogen, Carlsbad, CA) according to the manufacturer’s protocol. RNA was qualitatively and quantitatively assessed using a Nanodrop 2000C (Thermo Scientific, Waltham, MA).

### DNA methylation at CpG islands

Methylation of the 5’-regulatory region of the genome was analyzed by Reduced Representation Bisulfite Sequencing (RRBS) using the ABI 37370 (Applied Biosystems, Foster City, CA) with a 48 cm capillary array (Cold Spring Harbor Laboratory [CSHL] Core Facility).

### RNA sequencing

RNA sequencing analysis was conducted by Illumina sequencing (CSHL core facility). A False Discovery Rate (FDR) of 0.05 was used to determine differentially expressed genes. Genes selected were analyzed for log2 transformed fold change of expression level in tumor tissues compared to adjacent normal tissues. Those ≥0.05 were disregarded in the analyses.

### Statistical analysis

A trimming-and-retrieving alignment scheme, recently described by our group, was use for accurate global profiling of DNA methylation. This algorithm is specifically designed for the mapping of bisulfite converted reads from RRBS libraries. The methylation percentage calling is performed by counting methylated and unmethylated bases (and their ratio) on each site that is a C in the reference genome. This strategy ensures both accurate bisulfite-converted read alignment and methylation calling [21].

## Results

### Demographics of colon cancer subjects

The clinical metadata available for the WU samples were limited to age (at time of surgical resection of the tumor), sex (male/female) and race (AA or CA). DNA and RNA samples isolated from paired tumor and adjacent normal colon were prepared from 6 AA and 7 CA subjects. The average ages of the AA subjects (72.7±3.6) and CA subjects (62.4±6.3) were not significantly different. Similarly, the sex distribution was comparable between AA (3 female, 2 male, 1 unspecified) and CA (4 female, 3 male) patients.

### Increased genome-wide methylation in AA patients with colon cancer

**Fig 1:**
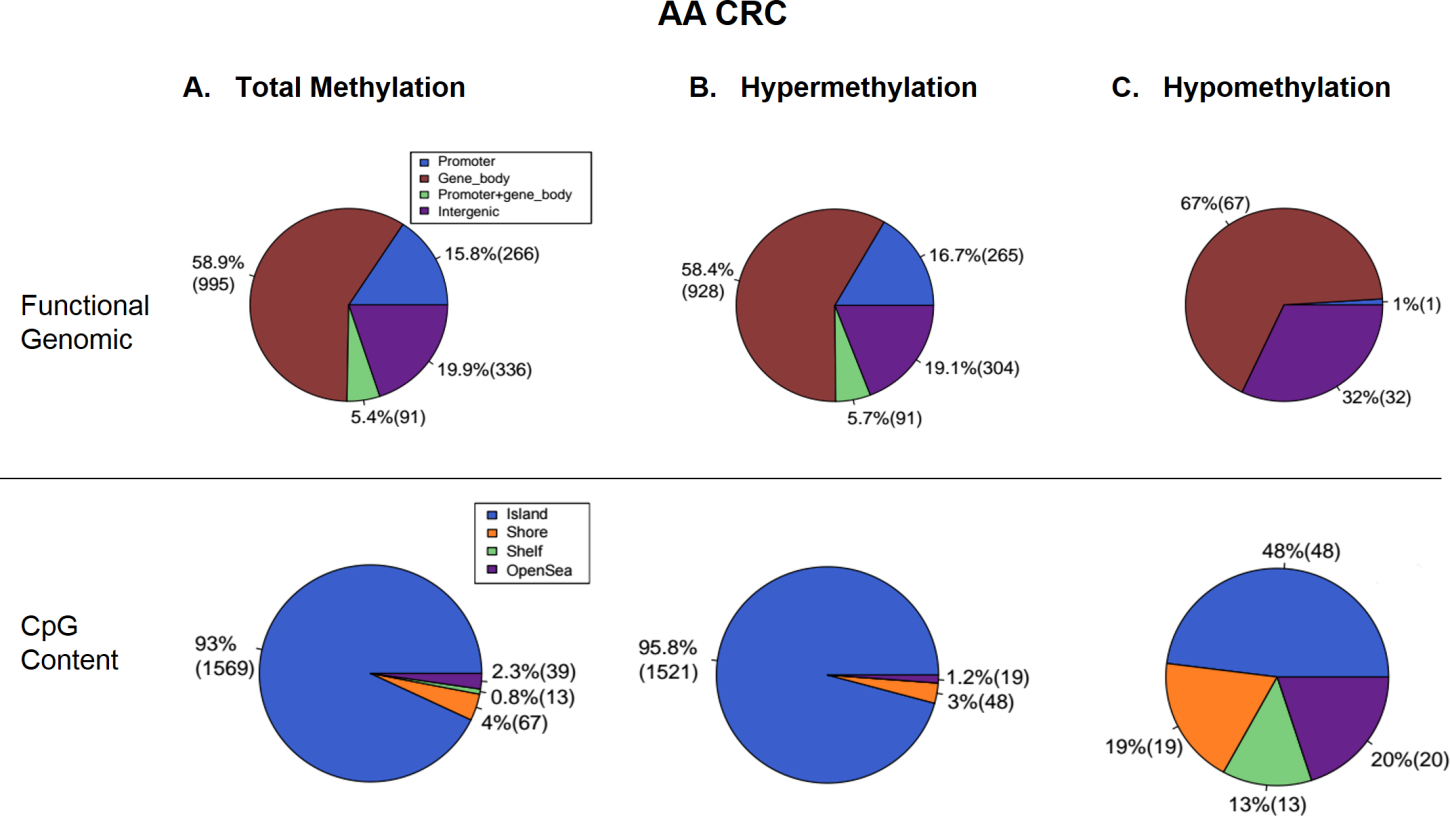
Aberrant methylation in tumors of AA patients with CRC. (A) A total of 27,059 methylated CpG sites were detected in 1,688 DMRs in AA CRC specimens. (B) 1,588 DMRs (94.1% of total DMRs) were hypermethylated, and (C) 100 DMRs (5.9%) were hypomethylated.

**Fig 2:**
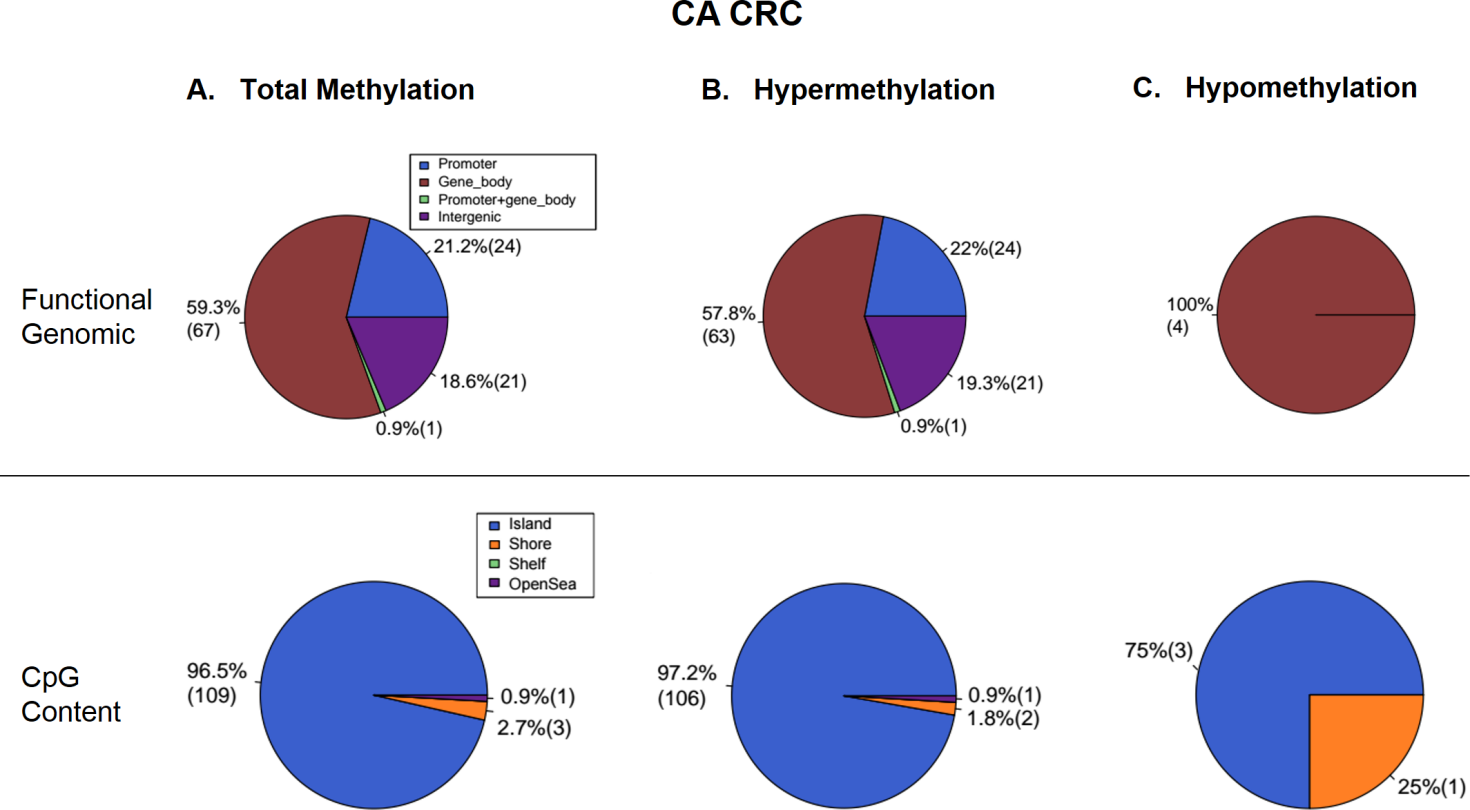
Aberrant methylation in tumors of CA patients with CRC. (A) 764 methylated CpG sites were identified across 113 DMRs in CA CRC specimens. (B) 109 DMRs (96.5% of total DMRs) were hypermethylated, and (C) 4 DMRs (3.5%) were hypomethylated. Aberrant methylation occurs in CA patients, but not to the same extent as for AA patients with CRC.

The total DNA methylation profiles of AA and CA CRC patients were examined using RRBS and analyzed using the BStool toolkit with a trimming-and-retrieving scheme. In total, there was a significantly greater number of DMR regions across the AA colon cancer samples compared to that found in samples of CA colon cancer patients. In DNA samples examined from AA patients, 27,059 methylated CpG sites were detected in 1,688 DMRs. 93% (1,569 sites) of methylated CpG sites was located within the island regions whereas 4% (67 sites) was within the shore regions. Furthermore, 15.8% (266 DMRs) of methylated DMRs was contained within the promoter, 5.4% (91 DMRs) spanned the promoter and gene body, and a majority (58.9%; 995 DMRs) existed within the gene body (Fig 1A). In comparison, 764 methylated CpG sites were identified across 113 DMRs of DNA from tumor samples of CA. 96.5% (109 sites) of methylated CpG sites was found within the island regions whereas 2.7% (3 sites) was within the shore regions. Furthermore, 21.2% (24 DMRs) of methylated DMRs was located within the promoter, 0.9% (1 DMR) spanned the promoter and gene body, and a majority (59.3%; 67 DMRs) existed within the gene body (Fig 2A). This represents a 35.4-fold and a 14.9-fold difference in methylated CpG sites and in DMRs in AA patients, respectively, with no observed differences in the percent composition of CpG sites (islands and shores) or DMRs (promoter, promoter and gene body, and gene body).

The rates of hyper- and hypomethylation were also analyzed for both sample sets. In DNA obtained from AA tumor samples, 1,588 DMRs were hypermethylated of which 95.8% (1,521 sites) was within CpG islands and 3% (48 sites) was within shores, and 16.7% (265 DMRs) was within the promoter, 5.7% (91 DMRs) spanned the promoter and gene body, and 58.4% (928 DMRs) was within the gene body (Fig 1B). In DNA from CA CRC specimens, 109 DMRs were hypermethylated of which 97.2% (106 sites) was within CpG islands and 1.8% (2 sites) was within shores, and 22% (24 DMRs) was within the promoter, 0.9% (1 DMR) spanned the promoter and gene body, and 57.8% (63 DMRs) was within the gene body (Fig 2B). Additionally, 100 DMRs were hypomethylated (5.9% of total DMRs) in AA CRC samples of which 48.8% (48 sites) was within CpG islands and 19% (19 sites) was within shores, and 1% (1 DMR) was within the promoter, 0% spanned the promoter and gene body, and 67% (67 DMRs) was within the gene body (Fig 1C). In contrast, 4 hypomethylated DMRs (3.5% of total DMRs) were observed in DNA obtained from CA tumor and compared to normal samples of which 75% (3 sites) was within CpG islands and 25% (1 site) was within shores, and 100% (4 DMRs) was within the gene body (Fig 2C).

### Genes are differentially methylated in AA CRC compared to CA CRC

Genes were ranked by their statistical significance of differential methylation for both AA and CA specimens compared to their respective matched normal tissue samples. An abridged list contains 23 hypermethylated genes and 4 hypomethylated genes (CACNA2D4;LRTM2, ESPNL, SECTM1, PCDH8) for AA tumor samples; whereas, 29 hypermethylated genes and 1 hypomethylated gene (BRSK2) are listed for the CA tumor samples (Table 1). These lists were then cross-referenced in order to illustrate shared differentially methylated genes in the AA and CA samples. 2 hypermethylated genes (CCDC178 and FLI1) were common between the two sample sets. Of note, the hypermethylation of CHL1, a member of neuronal cell adhesion molecules involved in neuronal development and synaptic plasticity, was found in AA CRC but not CA CRC.

**Table 1:** Annotated list of the highest differentially methylated genes for AA and CA CRC specimens, ranked by statistical significance.

African American Tumor Sample		Caucasian American Tumor Sample	
Gene	p-value	Gene	p-value
CACNA2D4;LRTM2	3.49E-25	QKI;CAHM	3.88E-29
FENDRR	4.77E-18	BRSK2	3.21E-17
APC2	8.43E-18	NDRG4	1.53E-16
GSC	1.16E-16	SDC2	2.73E-16
CHL1	4.64E-16	PHYHIPL	6.78E-15
KIAA1211L	1.26E-15	VWC2	2.01E-14
KCNA1	3.42E-15	GPR75-ASB3;GPR75	2.22E-14
ESPNL	5.85E-15	FBLL1	2.19E-13
LHX5	2.32E-14	LOC146880	4.14E-13
HOXA3	3.90E-14	FGF14	8.28E-12
LINC01398	3.31E-13	SH3GL3	8.48E-12
PTPRN2	3.53E-13	ESR1	3.28E-11
ECEL1	1.23E-12	FGF12	7.20E-11
GPR158;GPR158-AS1	1.88E-12	FLI1	3.01E-10
IFITM10	1.91E-12	ERICH1-AS1	4.03E-10
NELL1	2.39E-12	NRG3	5.61E-10
NPR3	2.40E-12	CCDC178	9.01E-10
MMD2	2.89E-12	CHST2	9.23E-10
C9orf50; NTMT1	9.34E-12	KCNG3	1.28E-09
CCDC178	1.05E-11	GDF6	6.50E-09
FLI1; SENCR	1.32E-11	HS3ST2	6.58E-09
C8orf34;LOC286189	2.42E-11	FLI1	8.38E-09
MDFI;MDFI	2.50E-11	ADAMTS2	1.57E-08
GUCY2D	3.02E-11	NKX6-2	2.02E-08
SECTM1	3.65E-11	RASA3	2.78E-08
GDF1;CERS1	4.15E-11	SLC6A2	3.13E-08
PCDH8;PCDH8	4.21E-11	CDH4	6.17E-08
		ALX4	7.18E-08
		FAM19A5	8.17E-08
		ANKRD13B	9.54E-08

### Racial disparity of differentially methylated microRNAs in CRC

Within the entire DNA methlyome of AA CRC specimens, 7 microRNAs were found to be hypermethylated compared to the respective matching adjacent normal tissue (Table 2) including miR-9-3p and miR-124-3p (S1 Fig). Likewise, two isoforms of miR-34 were the only miRNAs found to be hypermethylated in CA CRC samples. This study was unable to distinguish miRNAs that were significantly hypomethylated in either AA or CA tumors.

**Table 2:** Differently methylated miRNAs in AA and CA CRC specimens, ranked by statistical significance.

African American Tumor Sample		Caucasian American Tumor Sample	
miRNA	p-value	miRNA	p-value
miR-137HG;miR-2682	9.94E-11	miR-34b;miR-34c	2.51E-07
miR-9-3	2.01E-05		
miR-663A;miR-663AHG	5.84E-05		
miR-6130;RORB	6.65E-05		
miR-548AO	0.00016		
miR-124-3	0.000747316		

### Differential gene expression in AA CRC

**Fig 3:**
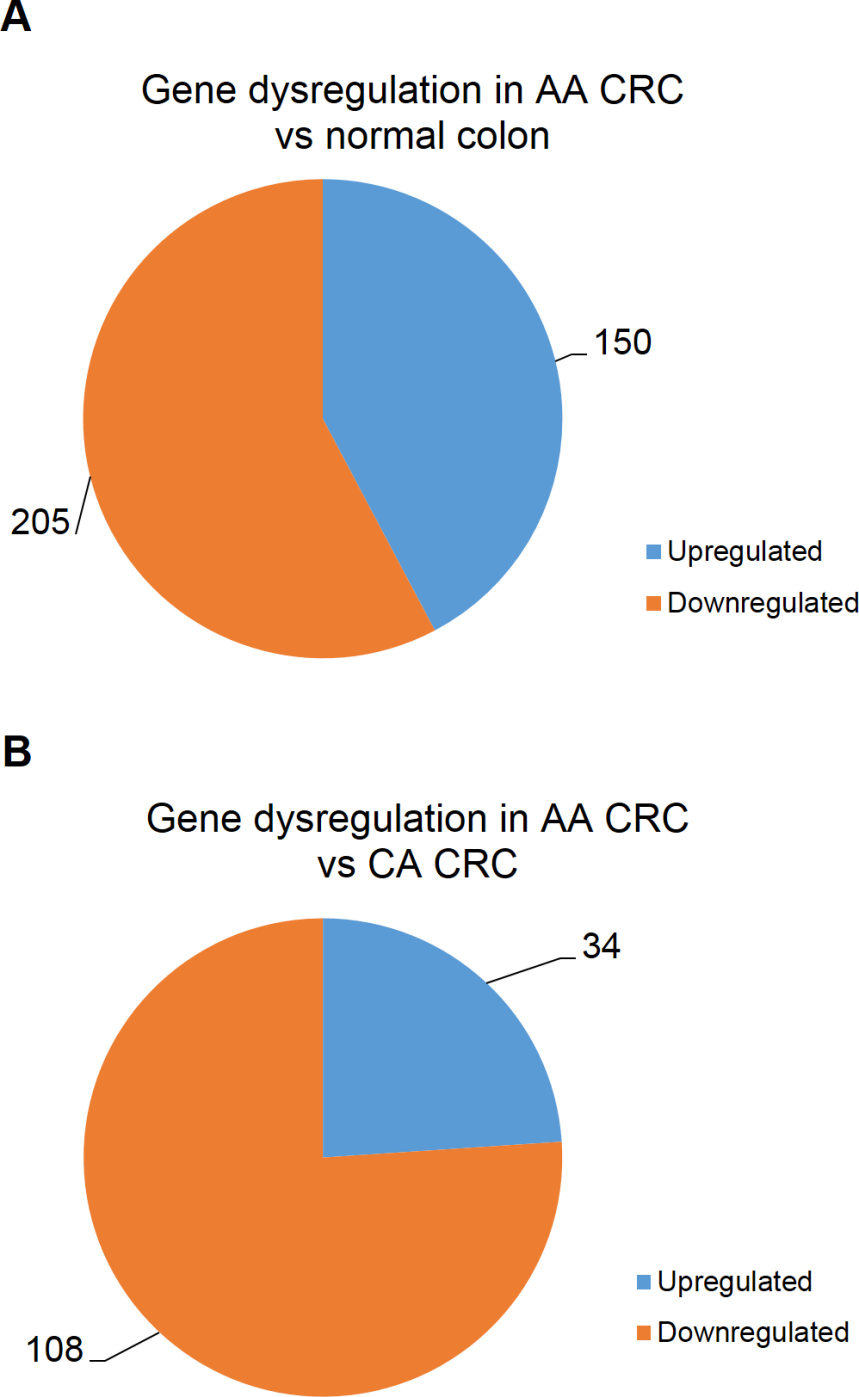
Dysregulation of gene expression is more prominent in AA compared to CA patients with CRC. (A) 205 genes were downregulated and 150 genes were upregulated in AA CRC compared to matching normal adjacent tissue. (B) 108 genes were downregulated and 34 genes were upregulated in AA CRC compared to CA CRC.

RNA sequencing was conducted using total RNA from the same patient CRC samples that were submitted for DNA methylation analysis. Analysis was first used to identify differentially expressed genes within the ethnic background for both AA and CA by evaluating CRC specimens against the respective matching adjacent normal tissues. For all analysis, only genes with p < 0.05 and a False Discovery Rate (FDR) < 0.05 were considered to be dysregulated. In AA CRC, 205 genes were downregulated (S1 Table) and 150 genes were upregulated (S2 Table) compared to the normal tissue (Fig 3A). Two miRNAs were reported within these differentially regulated genes; miR-4253 was upregulated and miR-3074 was downregulated. In the CA CRC specimens, 7 genes were upregulated (S3 Table) whereas no genes were reported to be downregulated when compared to normal adjacent tissue. Additionally, only SLCO4A1 and OXGR1 were upregulated in both AA and CA cases of CRC.

To evaluate the disparity of gene dysregulation between race, results obtained from AA CRC specimens were statistically analyzed against those results obtained from specimens of CA CRC. 108 genes were downregulated (S4 Table) and 34 genes were upregulated (S5 Table) in tumors of AA CRC patients versus CA CRC (Fig 3B). The top 15 downregulated and upregulated genes ranked by statistical significance are shown in Table 3 and Table 4, respectively. miR-1279 was found to be differentially upregulated in AA CRC compared to CA CRC, and was the only miRNA dysregulated between tumors of AA and CA CRC patients. Most strikingly, of the 108 genes downregulated in AA CRC tumors, 14 were ribosomal proteins including 10 members of the large 60S subunit (RPL7A, RPL8, RPL13, RPL13A, RPL18, RPL28, RPL29, RPL36, RPLP0, and RPLP1), 3 members of the small 40S subunit (RPS2, RPS15, and RPS19), and 1 mitochondrial ribosomal protein (MRPL12) of the large 39S subunit (Table 5). Additionally, two targets of miR-124-3p (POLR2Band CYP1B1) were upregulated.

**Table 3:** Annotated list of differentially downregulated genes in AA CRC compared to CA CRC, ranked by statistical significance.

Gene	Fold Change (log2)	p-value	FDR
RPL13	-2.650	6.91E-09	6.41E-05
HMGCS2	-4.244	1.55E-08	7.20E-05
MYH14	-2.591	4.15E-08	0.00013
TFF3	-3.381	1.13E-07	0.00023
CES2	-2.945	1.71E-07	0.00023
KRT19	-3.054	1.80E-07	0.00023
RPS2	-2.739	1.87E-07	0.00023
FAM3D	-3.447	2.02E-07	0.00023
RPL36	-2.350	2.39E-07	0.00025
RPL28	-2.351	4.69E-07	0.00037
C10orf99	-3.804	8.22E-07	0.00051
CDX1	-3.132	1.08E-06	0.00060
CHMP4B	-1.693	1.10E-06	0.00060
CXCL14	-2.548	1.20E-06	0.00062
YBX1	-1.786	1.65E-06	0.00081

**Table 4:** Annotated list of differentially upregulated genes in AA CRC compared to CA CRC, ranked by statistical significance.

Gene	Fold Change (log2)	p-value	FDR
THBS2	2.192	3.22E-07	0.00030
MNS1	1.920	4.73E-07	0.00037
BDNF-AS1	3.451	5.83E-07	0.00041
PCA3	3.530	6.24E-07	0.00041
DNM1P46	2.911	4.53E-06	0.00200
CYP1B1	3.149	1.15E-05	0.00357
OBSCN	2.066	1.61E-05	0.00428
BCAT1	2.980	3.63E-05	0.00823
RNF224	2.672	4.27E-05	0.00863
ZNF772	2.993	5.85E-05	0.01024
MAP4K4	1.452	6.52E-05	0.01070
EMB	2.660	8.19E-05	0.01226
MIR1279	1.563	8.65E-05	0.01249
SLC2A3	2.647	8.74E-05	0.01249
ZFHX4	3.021	0.00014	0.01745

**Table 5:** Ribosomal proteins are downregulated in AA CRC vs CA CRC.

Ribosomal Subunit	Protein	Extraribosomal Function(s) in *Homo Sapiens*	Expression Profile in CRC
	RPL7a	Rearranges with the TRK proto-oncogene thus encoding an oncoprotein consisting of the N-terminus of RPL7a fused to the receptor TRK domain [22]	Unknown
	RPL8	Unknown	Upregulated [23]
	RPL13	Unknown	Unknown; Overexpression in GI cancers leads to tumor growth and chemoresistance [24]
	RPL13a	Reduces inflammation via IFN-γ-activated inhibitor of translation (GAIT) complex [25, 26]	Downregulated [27]
	RPL18	Prevents PKR activation when associated with the ribosome;	Upregulated [23, 28]
60S		Overexpression may promote protein synthesis and cell growth through inhibition of PKR activity [29]	Downregulated [27]
	RPL28	Unknown	Upregulated [30]
			Downregulated [27]
	RPL29	Unknown; Knockdown in HT-29 cells induces overexpression of p21 and p53 and cell differentiation *in vitro* [31]	Upregulated [23]
			Downregulated [32]
	RPL36	Unknown	Hypermethylated [33]
	RPLP0	Regulates tumor progression, invasion, metastasis, and differentiation by influencing p21 and p53 expression [34, 35]	Upregulated [28, 34]
	RPLP1	Induces immortalization and proliferation in MEFs via activation of E2F transcription factors [36]	Upregulated with an accumulation of mutant p53 [36]
	RPS2	Implicated in the regulation of cell growth and proliferation [37, 38]	Unknown
40S	RPS15	Binds to MDM2 thus activating p53 and cell cycle arrest [39]	Unknown
	RPS19	*In vitro* knockdown 1) activates inflammation via p53-dependent TNF-α expression, and p38 MAPK expression leading to tumorigenesis, growth, and metastasis [40], and 2) decreases differentiation/maturation via GATA1 suppression [41]	Upregulated [23, 42]
Mito 39S	MRPL12	Binds to mitochondrial RNA polymerase POLRMT to promote transcription [43]	Unknown

## Discussion

Chronic colonic inflammation from inflammatory bowel diseases (IBDs) results in a well-recognized increased risk of colon carcinogenesis [44–47]. As stated by Rubin et al, “It has become increasingly clear that IBD is a polygenic, complex disorder with region- and ethnic-specific differences in genetic risk factors. In the past several years, progress in understanding the molecular basis of IBD has accelerated, beginning with the generation of animal models of colitis and progressing to the identification of specific genetic markers from candidate gene, gene linkage, and genome-wide association analyses” [48]. Therefore suppression of anti-inflammatory transcription factors, as with hypermethylation, would promote the development and progression of CRC. Here, we observed the aberrant hypermethylation of several genes that are implicated in anti-inflammatory mechanisms including NEL-Like 1 (NELL1) [49], Growth Differentiation Factor 1 (GDF1) [50], and Rho Guanine Nucleotide Exchange Factor (ARHGEF4) [51], and Integrin Alpha 4 (ITGA4) [52] in AA CRC but not CA CRC. Previous studies have demonstrated that ITGA4 is hypermethylated in inflamed colon tissue/colitis, and that the treatment of anti-ITGA4 antibodies further aggravate colitis by IL-1β, TNF-α, and IFN-γ recruitment [53]. Conversely, the treatment of ITGA4 antibodies in combination with conventional therapies alleviated colitis by suppression of IL-1β and iNOS in mouse models [54]. If these anti-inflammatory genes are constitutively hypermethylated in tumors of AA CRC patients, these proteins could potentially drive CRC initiation and progression and thereby serve as targets for pharmaceutical intervention and therapy.

Similarly, examining differentially methylated miRNAs and their effects on presumptive downstream gene targets is also critical in understanding the epigenetic variances responsible for increased incidence and mortality of CRC in African Americans. Here, we demonstrate that 7 miRNAs are hypermethylated in AA CRC specimens via RRBS. Two of these miRNAs have been implicated in the initiation of CRC; miR-9 and miR-124. miR-9 is downregulated in CRC resulting in the promotion of tumor survival and proliferation [55, 56]. Increased mortality rates are correlated with this inhibition [57]. miR-124 inhibits CRC progression *in vitro* and *in vivo* [58] and is suppressed in clinical specimens associated with IBD [59].

Analysis of altered downstream gene expression validates the observation of aberrant methylation. The upregulation of POLR2B and CYP1B1, 2 known targets of miR-124-3p [60], in AA CRC but not in CA CRC was the result of the hypermethylation of miR-124-3p. POLR2B, a DNA-dependent RNA polymerase, catalyzes the transcription of DNA to mRNA, microRNA, and small non-coding RNAs [61], and while POLR2B is not known to have a prominent role in the tumorigenesis and progression of cancer(s), a specific haplotype derived from single nucleotide polymorphisms (SNPs) is associated with an increased frequency of head and neck cancers [62]. CYP1B1, a member of the monooxygenase cytochrome P450 family, catalyzes drug metabolism and lipid synthesis. CYP1B1 localizes to the endoplasmic reticulum and metabolizes a variety of procarcinogens and xenobiotics [63]. Previous studies have demonstrated that CYP1B1 is overexpressed in colon cancers, and that the enzymatic activity is significantly higher in tumor specimens compared to normal colon tissue [64–66]. Interestingly, overexpression of CYP1B1 leads to the metabolism/biotransformation of docetaxel in *in vitro* models of breast cancer [67] and of flutamide, commonly prescribed in prostate cancer [68]; resulting in acquired chemotherapeutic resistance. Our preliminary data indicated that chemoresponse to 5-Fluorouracil was lower in an *in vivo* patient-derived mouse xenograft model of AA CRC but not in that of CA CRC (data not shown). The overexpression of CYP1B1 and consequential chemotherapeutic resistance in AA CRC is potentially a new avenue of investigation for defining effective treatments for CRC.

In the same vein, the dysregulation of RPs is a fundamental observation in nearly all carcinomas [69]. Identifying and characterizing disease-specific expression profile aids in the understanding and treatment of disease. Although RPs are primarily involved in translation, many have known secondary extraribosomal functions in cell proliferation, differentiation, tumorigenesis, and/or metastasis [69]. Even more roles are likely to exist as many RPs are not fully characterized. Conflicting reports have been published on the expression patterns of RPs in CRC. It may be intuitive that RPs would be constitutively upregulated in carcinomas as hyperactive translation contributes to uncontrolled proliferation, however several findings including our current data conclude that specific RPs are in fact downregulated in CRC. We have illustrated that 14 RPs are downregulated in AA CRC versus CA CRC including 10 members of the large 60S subunit, 3 members of the small 40S subunit, and 1 mitochondrial ribosomal protein of the large 39S subunit. Experimental design (i.e. specimen collection, sample size) and demographic data (age, sex, race/ethnicity) may contribute to contradictory results, but a biological explanation may lie within the extraribosomal functions of these RPs. For example, RPL13a regulates IFN-γ-activated inhibitor of translation complex-mediated inflammation, and silencing of RPL13a in macrophages results in the overexpression of inflammatory chemokines and systemic macrophage infiltration [25, 26]. *In vitro* knockdown of RPS19 in hematopoietic progenitor cells activates inflammation via increased p53-dependent TNF-α expression. Downregulated GATA1 expression mediated by p38 MAPK preventing hematopoietic differentiation was also observed, which may promote tumorigenesis, growth, and metastasis [40, 41]. Decreased RPL13a and RPS19 expression in AA CRC may contribute to inflammation, thus predisposing AAs to increased incidence and severity of CRC as previously discussed. Furthermore, the suppressed expression of ribosomal subunits, together with the hypermethylation of miR-124-3p and resulting upregulation of POLR2B, suggests a key role for aberrant mRNA transcription in the incidence of CRC for AA that is altogether unique from CA CRC patients. Overall, dysregulated transcription levels could result in increased cell proliferation and growth, migration/metastasis to secondary tissues, and acquired chemoresistance.

An important mechanism of tumorigenesis is epigenetic silencing of selected genes such as tumor suppressor or inflammation genes, by promoter methylation or by miRNAs. Here, our results demonstrate that hypermethylation of CHL1 was found to be significantly increased in the CRC tumors of AA as compared to CA patients. Hypermethylation of CHL1 is reportedly associated with its downregulation of gene expression. Downregulation of CHL1 has been implicated in several cancers including 48% of all CRC cases [70], and hypermethylation of CHL1 is associated with increased rates of deletions and MSIs in Iranian CRC specimens [71]. Still, the role of CHL1 in CRC is not fully understood or characterized. Interestingly, microRNA-182 is a negative regulator of CHL1 in human papillary thyroid carcinoma (PTC) with overexpression of miR-182 suppressing CHL1 and therefore promoting PTC cell proliferation and invasion [72]. Indeed, our lab has previously demonstrated overexpression of miR-182 in AA compared to CA CRC tumor samples [73].

These, and our previous findings, have provided potential candidates for addressing racial disparity in CRC. Overall, understanding dysregulation of methylation patterns in CRC will provide us with the tools for preventive or therapeutic interventions.

## Supporting Information

S1 Fig Unique miRNAs are aberrantly methylated in CRC. (A) miR-9-3p and (B) miR-124-3p, two miRNAs identified in our RRBS analysis, are independently verified as having significantly increased methylation in CRC according to the MethHC database of DNA Methylation and gene expression in Human Cancer (http://methhc.mbc.nctu.edu.tw/php/index.php).
(TIF)

S1 Table Downregulated genes in AA CRC compared normal adjacent tissue, ranked by statistical significance. (DOCX)

S2 Table Upregulated genes in AA CRC compared normal adjacent tissue, ranked by statistical significance. (DOCX)

S3 Table Upregulated genes in CA CRC compared normal adjacent tissue, ranked by statistical significance. (DOCX)

S4 Table Downregulated genes in AA CRC compared to CA CRC, ranked by statistical significance. (DOCX)

S5 Table Upregulated genes in AA CRC compared CA CRC, ranked by statistical significance. (DOCX)
