# Correction: Aberrant DNA Methylation: Implications in Racial Health Disparity

**DOI:** 10.1371/journal.pone.0158251

**Published:** 2016-06-21

**Authors:** Xuefeng Wang, Ping Ji, Yuanhao Zhang, Joseph F. LaComb, Xinyu Tian, Ellen Li, Jennie L. Williams

In the Methods section, a sentence from the DNA methylation at CpG islands subsection was inadvertently excluded. The complete, correct DNA methylation at CpG islands subsection should read as follows:

"Methylation of the 5'-regulatory region of the genome was analyzed by Reduced Representation Bisulfite Sequencing (RRBS) using the ABI 37370 (Applied Biosystems, Foster City, CA) with a 48 cm capillary array (Cold Spring Harbor Laboratory [CSHL] Core Facility). RRBS experiments were sequenced at an average depth of 100 million reads per sample, resulting in coverage ranging from 2.4–4 million CpG sites per sample (mean of 3 million CpG sites) and 0.7 million overlapped CpG sites across all samples."

Additionally, there is an error in the Funding section. The correct funding information is as follows: This study was supported by the National Institutes of Health—National Cancer Institute; http://www.cancer.gov/; grant number R01CA140487 to JLW and award number P20CA192994 to EL. Research was performed with additional assistance from CSHL Shared Resources, which are funded, in part, by the Cancer Center Support Grant 5P30CA045508. The funders had no role in study design, data collection and analysis, decision to publish, or preparation of the manuscript.
