# Supplementary figures and images for: Aberrant DNA Methylation: Implications in Racial Health Disparity
Source: PLoS One. 2016 Apr 25;11(4):e0153125. doi: 10.1371/journal.pone.0153125 (PMC4844165; doi:10.1371/journal.pone.0153125)

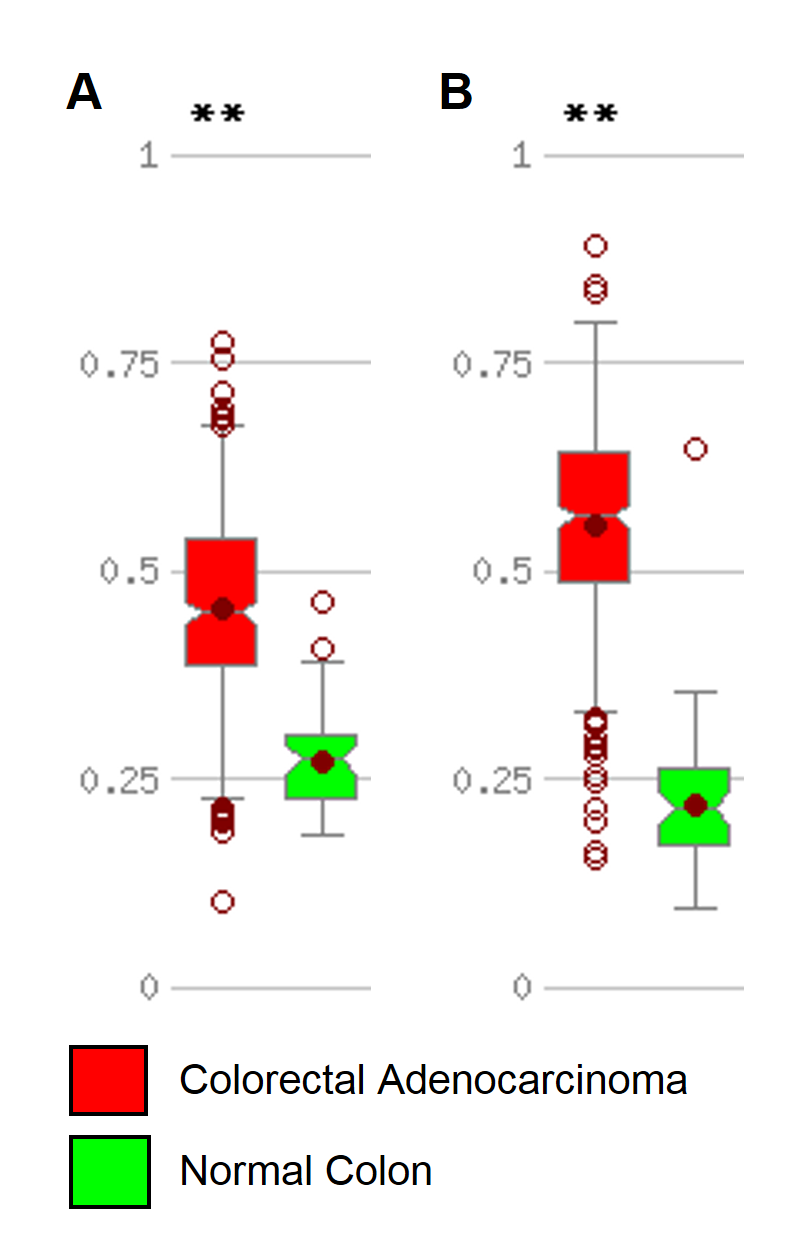

Supplement: S1 Fig — (A) miR-9-3p and (B) miR-124-3p, two miRNAs identified in our RRBS analysis, are independently verified as having significantly increased methylation in CRC according to the MethHC database of DNA Methylation and gene expression in Human Cancer (http://methhc.mbc.nctu.edu.tw/php/index.php). (TIF) [file pone.0153125.s001.tif]
