# Supplementary material for: Aberrant DNA Methylation: Implications in Racial Health Disparity
Source: PLoS One. 2016 Apr 25;11(4):e0153125. doi: 10.1371/journal.pone.0153125 (PMC4844165; doi:10.1371/journal.pone.0153125)
Supplement: S1 Table — (DOCX) [file pone.0153125.s002.docx]

**S1 Table. Downregulated genes in AA CRC compared normal adjacent tissue, ranked by statistical significance.**

| **Gene** | **Fold Change (log2)** | **p-value** | **FDR** |
| --- | --- | --- | --- |
| FCGBP | -5.760 | 1.42E-24 | 7.79E-21 |
| ITM2C | -4.054 | 2.07E-20 | 5.69E-17 |
| CA2 | -6.035 | 1.33E-18 | 2.44E-15 |
| ZG16 | -5.928 | 2.07E-17 | 2.84E-14 |
| MS4A12 | -5.666 | 1.33E-13 | 1.46E-10 |
| MIPOL1 | -3.814 | 5.94E-13 | 5.43E-10 |
| CEACAM7 | -5.007 | 3.17E-12 | 2.49E-09 |
| ZNRF1 | -3.421 | 2.30E-11 | 1.58E-08 |
| PADI2 | -3.956 | 4.71E-11 | 2.58E-08 |
| TBRG1 | -2.511 | 1.79E-10 | 8.95E-08 |
| NT5DC2 | -2.150 | 4.21E-10 | 1.92E-07 |
| SLC26A2 | -3.402 | 4.12E-09 | 1.61E-06 |
| ADH1C | -4.260 | 4.57E-09 | 1.67E-06 |
| CLU | -2.788 | 5.14E-09 | 1.74E-06 |
| KLF4 | -3.215 | 5.39E-09 | 1.74E-06 |
| SLC26A3 | -4.087 | 7.07E-09 | 2.15E-06 |
| AQP8 | -4.615 | 9.52E-09 | 2.75E-06 |
| LOC100131825 | -3.022 | 1.06E-08 | 2.90E-06 |
| CD177 | -4.344 | 1.72E-08 | 4.50E-06 |
| UGT2B17 | -4.078 | 1.98E-08 | 4.93E-06 |
| CA4 | -4.261 | 2.86E-08 | 6.83E-06 |
| GUCA2A | -4.444 | 4.19E-08 | 9.20E-06 |
| GPRIN1 | -2.596 | 1.25E-07 | 2.45E-05 |
| CBLL1 | -2.462 | 1.71E-07 | 3.13E-05 |
| FLVCR2 | -3.258 | 1.82E-07 | 3.23E-05 |
| ADAM22 | -2.940 | 2.22E-07 | 3.80E-05 |
| CDC42BPB | -2.672 | 2.55E-07 | 4.24E-05 |
| ADTRP | -3.748 | 2.69E-07 | 4.34E-05 |
| CTNS | -1.612 | 3.35E-07 | 4.96E-05 |
| CNN1 | -3.624 | 3.63E-07 | 5.10E-05 |
| BCAS1 | -3.131 | 4.95E-07 | 6.70E-05 |
| VSIG2 | -2.914 | 6.00E-07 | 7.83E-05 |
| CHP2 | -3.529 | 9.95E-07 | 0.00012 |
| TSPAN1 | -3.185 | 1.04E-06 | 0.00012 |
| PHGR1 | -3.760 | 1.08E-06 | 0.00012 |
| CACNA1H | -2.573 | 1.62E-06 | 0.00018 |
| SLC17A1 | -3.492 | 2.04E-06 | 0.00022 |
| ABHD11 | -2.552 | 2.40E-06 | 0.00026 |
| SLIT3 | -2.969 | 2.59E-06 | 0.00027 |
| MAPK7 | -1.840 | 2.71E-06 | 0.00028 |
| DHRS11 | -3.015 | 2.91E-06 | 0.00030 |
| MAZ | -1.946 | 3.05E-06 | 0.00030 |
| THBS1 | -1.973 | 4.13E-06 | 0.00040 |
| CHGA | -3.372 | 4.40E-06 | 0.00041 |
| MYH15 | -2.617 | 5.94E-06 | 0.00054 |
| TPM2 | -2.309 | 6.12E-06 | 0.00054 |
| TMEM120B | -2.460 | 6.56E-06 | 0.00056 |
| GTF2A2 | -2.023 | 7.99E-06 | 0.00067 |
| POLR3H | -1.696 | 8.41E-06 | 0.00070 |
| HMGCS2 | -3.476 | 9.79E-06 | 0.00080 |
| NMT1 | -2.299 | 1.09E-05 | 0.00088 |
| BBIP1 | -1.881 | 1.12E-05 | 0.00089 |
| PLK1 | -1.989 | 1.17E-05 | 0.00092 |
| PIGR | -3.084 | 1.44E-05 | 0.00111 |
| PPID | -1.740 | 1.48E-05 | 0.00112 |
| PIGH | -1.939 | 1.67E-05 | 0.00123 |
| PKIB | -3.475 | 1.72E-05 | 0.00124 |
| CSRP1 | -1.847 | 1.74E-05 | 0.00124 |
| PACSIN1 | -2.976 | 1.84E-05 | 0.00126 |
| NFRKB | -2.111 | 1.90E-05 | 0.00128 |
| KRI1 | -1.893 | 2.02E-05 | 0.00132 |
| GCNT3 | -2.776 | 2.03E-05 | 0.00132 |
| RECQL5 | -1.795 | 2.16E-05 | 0.00139 |
| NCAPH2 | -1.660 | 2.51E-05 | 0.00159 |
| NBPF7 | -2.961 | 2.78E-05 | 0.00173 |
| SLC5A10 | -1.985 | 3.71E-05 | 0.00228 |
| EHMT2 | -1.778 | 3.81E-05 | 0.00230 |
| CKB | -2.761 | 3.90E-05 | 0.00233 |
| CREB3 | -1.578 | 4.37E-05 | 0.00255 |
| NDE1 | -2.202 | 4.59E-05 | 0.00264 |
| CHRNE | -1.403 | 4.65E-05 | 0.00264 |
| MOCS1 | -1.732 | 4.68E-05 | 0.00264 |
| ANKRD52 | -2.216 | 5.06E-05 | 0.00283 |
| MUC2 | -2.646 | 5.20E-05 | 0.00284 |
| IL24 | -1.943 | 5.21E-05 | 0.00284 |
| SLC4A4 | -2.683 | 5.23E-05 | 0.00284 |
| C22orf15 | -2.359 | 5.47E-05 | 0.00287 |
| LGALS3 | -1.586 | 5.53E-05 | 0.00287 |
| TNFRSF14 | -2.007 | 5.54E-05 | 0.00287 |
| BEST1 | -1.497 | 5.94E-05 | 0.00302 |
| SRI | -2.198 | 6.33E-05 | 0.00316 |
| TP53INP2 | -2.373 | 6.76E-05 | 0.00331 |
| NPHS2 | -2.495 | 7.05E-05 | 0.00342 |
| ACTG2 | -2.724 | 7.40E-05 | 0.00353 |
| ZNF48 | -2.259 | 7.64E-05 | 0.00361 |
| AHCYL2 | -2.621 | 7.84E-05 | 0.00365 |
| LOC100652768 | -1.234 | 7.85E-05 | 0.00365 |
| ACSF2 | -2.611 | 8.72E-05 | 0.00402 |
| SKIV2L2 | -1.522 | 8.82E-05 | 0.00403 |
| C9orf100 | -1.172 | 9.77E-05 | 0.00439 |
| TSTD2 | -1.692 | 0.000102541 | 0.00457 |
| LLGL1 | -1.880 | 0.000103924 | 0.00460 |
| C1orf123 | -1.881 | 0.000117033 | 0.00514 |
| FAM55D | -2.776 | 0.000121834 | 0.00520 |
| COQ6 | -1.733 | 0.000122412 | 0.00520 |
| MIB2 | -1.725 | 0.000127049 | 0.00532 |
| SLC25A25 | -2.154 | 0.000132111 | 0.00549 |
| KIF13B | -2.315 | 0.000139618 | 0.00576 |
| ACAA2 | -2.282 | 0.000141079 | 0.00577 |
| ATP5G3 | -1.761 | 0.00015144 | 0.00615 |
| DES | -2.684 | 0.000160213 | 0.00646 |
| IGJ | -2.530 | 0.000169738 | 0.00675 |
| TSPAN3 | -1.279 | 0.000170992 | 0.00675 |
| FAM3D | -2.668 | 0.000186969 | 0.00711 |
| HPGD | -2.233 | 0.000189834 | 0.00713 |
| TAGLN | -1.609 | 0.000196754 | 0.00734 |
| SRPR | -1.282 | 0.000201256 | 0.00746 |
| PTK2B | -2.256 | 0.00020612 | 0.00752 |
| LOC100507053 | -2.541 | 0.000208369 | 0.00752 |
| C17orf76-AS1 | -1.501 | 0.00022201 | 0.00796 |
| MYL6 | -1.318 | 0.000237202 | 0.00839 |
| GSN | -1.701 | 0.000293763 | 0.01007 |
| C17orf81 | -1.425 | 0.000296972 | 0.01012 |
| GPR68 | -2.002 | 0.00031052 | 0.01051 |
| ERI2 | -1.971 | 0.000368953 | 0.01226 |
| LOC645513 | -1.550 | 0.000382848 | 0.01250 |
| PLA2G2A | -2.464 | 0.000397781 | 0.01283 |
| APBA3 | -1.896 | 0.000412805 | 0.01308 |
| AKR1B10 | -2.841 | 0.000418673 | 0.01312 |
| DLEC1 | -1.744 | 0.000447234 | 0.01386 |
| KRT20 | -2.501 | 0.000476748 | 0.01461 |
| CA12 | -1.855 | 0.000484845 | 0.01477 |
| TNK2 | -1.883 | 0.000490471 | 0.01486 |
| TCTN1 | -1.738 | 0.000509665 | 0.01536 |
| CCDC152 | -1.681 | 0.000536654 | 0.01582 |
| HPCA | -2.267 | 0.00053936 | 0.01582 |
| MAGI2 | -2.358 | 0.000552139 | 0.01602 |
| SLC22A18 | -2.359 | 0.000563143 | 0.01610 |
| ETHE1 | -2.234 | 0.000563702 | 0.01610 |
| BCKDHA | -1.821 | 0.000620077 | 0.01743 |
| HSPG2 | -1.956 | 0.00063659 | 0.01763 |
| CPT1A | -1.544 | 0.000685835 | 0.01868 |
| KLF9 | -1.464 | 0.000687053 | 0.01868 |
| CLTB | -1.666 | 0.000687441 | 0.01868 |
| SPINT1 | -1.246 | 0.000689993 | 0.01868 |
| FTH1 | -1.498 | 0.00071484 | 0.01902 |
| ACADVL | -1.585 | 0.000730028 | 0.01916 |
| CDHR5 | -1.857 | 0.000735337 | 0.01921 |
| SIDT2 | -1.325 | 0.000747281 | 0.01933 |
| MYO1A | -2.331 | 0.000850738 | 0.02159 |
| SELENBP1 | -2.493 | 0.000853864 | 0.02159 |
| SLC29A2 | -1.641 | 0.000956645 | 0.02374 |
| MYL9 | -1.912 | 0.000994884 | 0.02447 |
| SQRDL | -2.137 | 0.001006673 | 0.02454 |
| LGALS4 | -2.279 | 0.001027741 | 0.02494 |
| HIGD2A | -1.704 | 0.001084236 | 0.02608 |
| LOC100289255 | -2.541 | 0.001099272 | 0.02624 |
| CD63 | -1.322 | 0.00111297 | 0.02643 |
| POLR2C | -1.383 | 0.001139569 | 0.02694 |
| CES2 | -1.922 | 0.001234767 | 0.02882 |
| PI4K2A | -1.873 | 0.001241406 | 0.02882 |
| C9orf129 | -1.749 | 0.001284123 | 0.02947 |
| RASL12 | -2.351 | 0.001306505 | 0.02979 |
| TNFRSF13B | -2.632 | 0.001369585 | 0.03057 |
| LOC100505633 | -2.252 | 0.001370925 | 0.03057 |
| METTL7A | -2.410 | 0.001434436 | 0.03157 |
| GLTP | -2.041 | 0.001450143 | 0.03157 |
| ZSCAN2 | -1.571 | 0.00145027 | 0.03157 |
| TRIM14 | -1.751 | 0.00146548 | 0.03177 |
| MYO7B | -1.944 | 0.001612652 | 0.03373 |
| NOXA1 | -1.873 | 0.001613112 | 0.03373 |
| SPINK4 | -2.668 | 0.001712079 | 0.03499 |
| RELL1 | -1.896 | 0.001717883 | 0.03499 |
| NHSL1 | -1.728 | 0.001722526 | 0.03499 |
| MBNL1 | -1.571 | 0.001749013 | 0.03540 |
| BTNL3 | -1.824 | 0.001759797 | 0.03547 |
| STARD10 | -1.892 | 0.001791033 | 0.03585 |
| ATP5S | -1.832 | 0.001886227 | 0.03749 |
| COX4I1 | -1.615 | 0.001953788 | 0.03814 |
| HPS3 | -1.733 | 0.002013294 | 0.03878 |
| ACOT11 | -2.190 | 0.00205709 | 0.03918 |
| PRR13 | -1.831 | 0.002086036 | 0.03952 |
| FUCA1 | -1.952 | 0.002094823 | 0.03952 |
| ST14 | -1.614 | 0.002096504 | 0.03952 |
| LIMD2 | -1.254 | 0.002148525 | 0.03995 |
| MIR3074 | -2.141 | 0.002217682 | 0.04082 |
| NLRP9 | -1.744 | 0.002232925 | 0.04096 |
| CNOT7 | -1.609 | 0.002252366 | 0.04118 |
| CYBA | -1.834 | 0.002352045 | 0.04214 |
| MVP | -1.818 | 0.00235845 | 0.04214 |
| SGPP2 | -1.668 | 0.002385126 | 0.04214 |
| SEPP1 | -1.626 | 0.002412036 | 0.04227 |
| ACVRL1 | -1.987 | 0.002430256 | 0.04232 |
| TAF10 | -1.294 | 0.002449132 | 0.04251 |
| LITAF | -1.263 | 0.002519124 | 0.04359 |
| MYL5 | -1.657 | 0.002597658 | 0.04434 |
| YPEL1 | -1.254 | 0.002607262 | 0.04434 |
| CEACAM1 | -2.099 | 0.00261411 | 0.04434 |
| SHMT1 | -1.464 | 0.002618939 | 0.04434 |
| CABYR | -1.627 | 0.002636187 | 0.04441 |
| PEX26 | -1.896 | 0.002668289 | 0.04476 |
| TYMS | -1.147 | 0.002680365 | 0.04482 |
| ARL14 | -2.168 | 0.002820241 | 0.04645 |
| TESK1 | -1.914 | 0.002844906 | 0.04670 |
| CLDN7 | -1.661 | 0.002889964 | 0.04690 |
| C19orf21 | -2.084 | 0.002924115 | 0.04694 |
| ANXA11 | -1.624 | 0.00292691 | 0.04694 |
| PP7080 | -1.344 | 0.002947742 | 0.04700 |
| TRPT1 | -1.493 | 0.002984564 | 0.04720 |
| DPT | -1.555 | 0.003019187 | 0.04722 |
| ADAMDEC1 | -2.400 | 0.003063268 | 0.04773 |
| VAMP8 | -1.213 | 0.00315259 | 0.04871 |
| LPIN3 | -1.806 | 0.003161923 | 0.04872 |
| PRCD | -1.404 | 0.003209808 | 0.04918 |
| LTBP3 | -1.228 | 0.003229616 | 0.04934 |
