# Supplementary material for: Aberrant DNA Methylation: Implications in Racial Health Disparity
Source: PLoS One. 2016 Apr 25;11(4):e0153125. doi: 10.1371/journal.pone.0153125 (PMC4844165; doi:10.1371/journal.pone.0153125)
Supplement: S2 Table — (DOCX) [file pone.0153125.s003.docx]

**S2 Table. Upregulated genes in AA CRC compared normal adjacent tissue, ranked by statistical significance.**

| **Gene** | **Fold Change (log2)** | **p-value** | **FDR** |
| --- | --- | --- | --- |
| FSIP2 | 2.684 | 3.54E-11 | 2.16E-08 |
| LOC100127888 | 2.701 | 1.79E-09 | 7.54E-07 |
| MLK7-AS1 | 2.396 | 3.16E-08 | 7.23E-06 |
| OXGR1 | 4.331 | 8.80E-08 | 1.86E-05 |
| KIAA0895 | 3.199 | 1.21E-07 | 2.45E-05 |
| MCM3AP | 2.413 | 1.45E-07 | 2.75E-05 |
| TPT1-AS1 | 1.705 | 2.86E-07 | 4.38E-05 |
| C8orf39 | 2.661 | 2.87E-07 | 4.38E-05 |
| VCAN | 3.042 | 3.44E-07 | 4.96E-05 |
| COL11A1 | 4.136 | 5.01E-07 | 6.70E-05 |
| TOP2A | 2.594 | 6.73E-07 | 8.58E-05 |
| LY6G6F | 3.458 | 8.13E-07 | 0.00010 |
| SCD | 2.525 | 1.01E-06 | 0.00012 |
| LOC641518 | 3.164 | 4.33E-06 | 0.00041 |
| TRPM2 | 3.011 | 4.37E-06 | 0.00041 |
| LINC00312 | 2.947 | 5.99E-06 | 0.00054 |
| FAM63A | 3.120 | 6.38E-06 | 0.00056 |
| ZFP41 | 3.038 | 1.48E-05 | 0.00112 |
| FMR1-AS1 | 1.998 | 1.72E-05 | 0.00124 |
| MAPK15 | 2.669 | 1.76E-05 | 0.00124 |
| STK17A | 1.479 | 1.81E-05 | 0.00125 |
| DLEU7 | 3.029 | 1.91E-05 | 0.00128 |
| PKMYT1 | 1.423 | 2.52E-05 | 0.00159 |
| KHK | 2.360 | 3.80E-05 | 0.00230 |
| MIR4253 | 2.117 | 4.16E-05 | 0.00245 |
| ANKRD36BP1 | 1.982 | 5.38E-05 | 0.00287 |
| TAF9B | 1.560 | 5.49E-05 | 0.00287 |
| LOC100652999 | 1.885 | 5.63E-05 | 0.00289 |
| SLCO4A1 | 2.198 | 6.04E-05 | 0.00304 |
| LOC100507346 | 2.085 | 6.49E-05 | 0.00321 |
| DPY19L2P2 | 2.804 | 7.11E-05 | 0.00342 |
| FBP2 | 1.910 | 9.21E-05 | 0.00417 |
| TOMM34 | 2.010 | 0.00011836 | 0.00515 |
| C3orf24 | 1.642 | 0.000119518 | 0.00516 |
| RAD21-AS1 | 2.057 | 0.000125454 | 0.00529 |
| MLLT4-AS1 | 2.714 | 0.000168196 | 0.00673 |
| LOC100131626 | 1.806 | 0.000172684 | 0.00677 |
| LOC100294362 | 1.706 | 0.00017571 | 0.00684 |
| LOC100128191 | 1.775 | 0.000177827 | 0.00687 |
| LOC100506994 | 2.019 | 0.00018241 | 0.00700 |
| ZFHX3 | 1.538 | 0.000187858 | 0.00711 |
| C10orf55 | 1.726 | 0.000203563 | 0.00749 |
| PRKDC | 1.861 | 0.000207799 | 0.00752 |
| ATP11A | 2.726 | 0.000236122 | 0.00839 |
| PTPRO | 2.960 | 0.000247511 | 0.00867 |
| TLX1NB | 2.968 | 0.000248232 | 0.00867 |
| GRIP2 | 1.422 | 0.000279254 | 0.00969 |
| AKIRIN2-AS1 | 1.255 | 0.000281417 | 0.00971 |
| ERCC1 | 1.619 | 0.000321245 | 0.01081 |
| CANX | 1.255 | 0.000344214 | 0.01151 |
| TMED10P1 | 1.481 | 0.00037772 | 0.01241 |
| CENPF | 1.965 | 0.000377728 | 0.01241 |
| ANAPC10 | 2.107 | 0.000390097 | 0.01266 |
| GCFC2 | 1.550 | 0.000412317 | 0.01308 |
| TPX2 | 2.042 | 0.000413656 | 0.01308 |
| DUSP16 | 1.224 | 0.00041493 | 0.01308 |
| LCTL | 1.223 | 0.000446018 | 0.01386 |
| PRUNE2 | 1.727 | 0.000476733 | 0.01461 |
| LOC100130197 | 1.800 | 0.000521916 | 0.01563 |
| LOC100616668 | 1.443 | 0.000524232 | 0.01563 |
| SYTL4 | 1.646 | 0.000531171 | 0.01575 |
| LOC100505933 | 2.038 | 0.000548842 | 0.01601 |
| KCNQ1OT1 | 1.121 | 0.000561266 | 0.01610 |
| CNNM3 | 1.234 | 0.000577649 | 0.01642 |
| SPAG17 | 1.281 | 0.000582472 | 0.01647 |
| RSF1 | 1.840 | 0.00062269 | 0.01743 |
| IDI2-AS1 | 1.370 | 0.000628059 | 0.01749 |
| PRSS54 | 1.695 | 0.000693233 | 0.01868 |
| AFAP1L2 | 1.765 | 0.000694934 | 0.01868 |
| CHD6 | 1.899 | 0.000709964 | 0.01900 |
| PRR3 | 1.678 | 0.000720356 | 0.01902 |
| FLJ38109 | 1.652 | 0.000721116 | 0.01902 |
| GLIPR1 | 1.084 | 0.000742441 | 0.01930 |
| CLDN18 | 2.581 | 0.000825182 | 0.02125 |
| ZNF337 | 1.581 | 0.000843709 | 0.02159 |
| C20orf132 | 1.403 | 0.000857271 | 0.02159 |
| LOC286190 | 1.484 | 0.000860584 | 0.02159 |
| DPH3P1 | 1.527 | 0.000862965 | 0.02159 |
| SLC3A1 | 1.302 | 0.000866048 | 0.02159 |
| RBP5 | 2.122 | 0.000987855 | 0.02441 |
| LOC100507156 | 1.818 | 0.001002104 | 0.02454 |
| HSP90AA1 | 1.134 | 0.00106718 | 0.02579 |
| TSC22D1-AS1 | 1.725 | 0.001100386 | 0.02624 |
| LOC283104 | 1.616 | 0.001150144 | 0.02708 |
| ZRANB2-AS1 | 1.207 | 0.001237834 | 0.02882 |
| STT3B | 1.511 | 0.001245237 | 0.02882 |
| BACE1 | 1.364 | 0.001282339 | 0.02947 |
| NUP93 | 2.386 | 0.001308724 | 0.02979 |
| IL28RA | 2.142 | 0.001329491 | 0.03013 |
| IGFBP5 | 1.418 | 0.001337805 | 0.03020 |
| SULF1 | 1.865 | 0.001368009 | 0.03057 |
| DZIP1 | 2.506 | 0.001408397 | 0.03125 |
| PHKA1 | 2.649 | 0.001412828 | 0.03125 |
| PVRIG | 1.873 | 0.001440547 | 0.03157 |
| IL7 | 1.529 | 0.001471569 | 0.03178 |
| GBP7 | 2.099 | 0.001481738 | 0.03187 |
| FAM69A | 1.164 | 0.001506003 | 0.03227 |
| HSP90AB1 | 1.348 | 0.001540898 | 0.03289 |
| LOC100506540 | 1.365 | 0.001576309 | 0.03351 |
| C5orf34 | 1.968 | 0.001594395 | 0.03373 |
| SERPINA5 | 2.438 | 0.001622585 | 0.03373 |
| DCAF13 | 1.599 | 0.001623611 | 0.03373 |
| PPP6R3 | 2.719 | 0.001686787 | 0.03491 |
| CEP290 | 1.321 | 0.001698022 | 0.03499 |
| LGALS8-AS1 | 1.531 | 0.001715199 | 0.03499 |
| BACE1-AS | 1.929 | 0.001765355 | 0.03547 |
| MAP3K14 | 1.547 | 0.001797398 | 0.03585 |
| LOC100009676 | 1.610 | 0.001901493 | 0.03765 |
| LRRC37A | 1.519 | 0.001912825 | 0.03765 |
| CMTM6 | 1.553 | 0.001915293 | 0.03765 |
| ATAD2 | 2.052 | 0.001949109 | 0.03814 |
| KRT10 | 1.757 | 0.001993243 | 0.03877 |
| FNBP1L | 1.336 | 0.002003398 | 0.03878 |
| CHD4 | 1.182 | 0.002024826 | 0.03878 |
| PGM5P2 | 2.020 | 0.002027318 | 0.03878 |
| CBX3 | 1.561 | 0.002028979 | 0.03878 |
| LOC100131320 | 1.615 | 0.002106963 | 0.03958 |
| RACGAP1 | 1.905 | 0.002136394 | 0.03993 |
| FAIM3 | 2.383 | 0.002140198 | 0.03993 |
| GAPDHS | 1.209 | 0.002157775 | 0.03998 |
| SPP1 | 2.401 | 0.002174728 | 0.04016 |
| PPAPDC2 | 1.502 | 0.002262771 | 0.04121 |
| COL4A1 | 1.708 | 0.002268852 | 0.04121 |
| PRKRA | 1.495 | 0.002321639 | 0.04203 |
| FBXO22-AS1 | 1.169 | 0.002371169 | 0.04214 |
| ATF5 | 1.374 | 0.002387081 | 0.04214 |
| PRR19 | 1.025 | 0.002396684 | 0.04214 |
| SLC16A4 | 1.610 | 0.002397056 | 0.04214 |
| TGS1 | 1.598 | 0.002422212 | 0.04231 |
| LOC100128531 | 1.534 | 0.002552432 | 0.04392 |
| LOC340544 | 1.492 | 0.002554044 | 0.04392 |
| LOC100288077 | 2.637 | 0.002601067 | 0.04434 |
| C1orf192 | 1.259 | 0.00263971 | 0.04441 |
| ARHGAP11A | 1.968 | 0.002705135 | 0.04510 |
| ACAD11 | 1.973 | 0.002765478 | 0.04597 |
| CHI3L1 | 2.475 | 0.002781684 | 0.04610 |
| EPB41L5 | 1.855 | 0.002802332 | 0.04630 |
| GCFC1 | 1.705 | 0.002854318 | 0.04670 |
| KAZN | 1.044 | 0.002863733 | 0.04670 |
| ZNF510 | 1.958 | 0.002869341 | 0.04670 |
| GCFC1-AS1 | 1.285 | 0.002900307 | 0.04693 |
| ARV1 | 1.997 | 0.002912675 | 0.04694 |
| KIAA1199 | 1.385 | 0.00294199 | 0.04700 |
| STK38L | 1.432 | 0.002956123 | 0.04700 |
| LOC647979 | 1.235 | 0.002986591 | 0.04720 |
| KAAG1 | 2.263 | 0.003001651 | 0.04720 |
| FCGR3A | 1.751 | 0.003022006 | 0.04722 |
| LOC440297 | 1.873 | 0.003111424 | 0.04835 |
| DCBLD1 | 1.335 | 0.003149113 | 0.04871 |
| EIF2AK2 | 1.384 | 0.003177147 | 0.04881 |
