# Supplementary material for: Aberrant DNA Methylation: Implications in Racial Health Disparity
Source: PLoS One. 2016 Apr 25;11(4):e0153125. doi: 10.1371/journal.pone.0153125 (PMC4844165; doi:10.1371/journal.pone.0153125)
Supplement: S3 Table — (DOCX) [file pone.0153125.s004.docx]

**S3 Table. Upregulated genes in CA CRC compared normal adjacent tissue, ranked by statistical significance.**

| **Gene** | **Fold Change (log2)** | **p-value** | **FDR** |
| --- | --- | --- | --- |
| SLCO4A1 | 2.807 | 2.74E-09 | 2.33E-05 |
| EVX1 | 2.721 | 1.07E-06 | 0.0034 |
| TGFBI | 2.106 | 1.21E-06 | 0.0034 |
| DPEP1 | 2.607 | 2.58E-06 | 0.0055 |
| SSTR5 | 2.494 | 6.15E-06 | 0.0105 |
| CLDN2 | 2.321 | 1.18E-05 | 0.0164 |
| OXGR1 | 2.409 | 1.35E-05 | 0.0164 |
