# Supplementary material for: Aberrant DNA Methylation: Implications in Racial Health Disparity
Source: PLoS One. 2016 Apr 25;11(4):e0153125. doi: 10.1371/journal.pone.0153125 (PMC4844165; doi:10.1371/journal.pone.0153125)
Supplement: S4 Table — (DOCX) [file pone.0153125.s005.docx]

**S4 Table. Downregulated genes in AA CRC compared to CA CRC, ranked by statistical significance.**

| **Gene** | **Fold Change (log2)** | **p-value** | **FDR** |
| --- | --- | --- | --- |
| RPL13 | -2.650 | 6.91E-09 | 6.41E-05 |
| HMGCS2 | -4.244 | 1.55E-08 | 7.20E-05 |
| MYH14 | -2.591 | 4.15E-08 | 0.00013 |
| TFF3 | -3.381 | 1.13E-07 | 0.00023 |
| CES2 | -2.945 | 1.71E-07 | 0.00023 |
| KRT19 | -3.054 | 1.80E-07 | 0.00023 |
| RPS2 | -2.739 | 1.87E-07 | 0.00023 |
| FAM3D | -3.447 | 2.02E-07 | 0.00023 |
| RPL36 | -2.350 | 2.39E-07 | 0.00025 |
| RPL28 | -2.351 | 4.69E-07 | 0.00037 |
| C10orf99 | -3.804 | 8.22E-07 | 0.00051 |
| CDX1 | -3.132 | 1.08E-06 | 0.00060 |
| CHMP4B | -1.693 | 1.10E-06 | 0.00060 |
| CXCL14 | -2.548 | 1.20E-06 | 0.00062 |
| YBX1 | -1.786 | 1.65E-06 | 0.00081 |
| LLGL2 | -3.112 | 3.81E-06 | 0.00177 |
| LAMP1 | -2.039 | 5.71E-06 | 0.00241 |
| MPST | -3.518 | 6.85E-06 | 0.00276 |
| PPDPF | -2.902 | 7.12E-06 | 0.00276 |
| ZG16 | -3.682 | 1.00E-05 | 0.00349 |
| EEF1D | -1.807 | 1.03E-05 | 0.00349 |
| GALE | -3.002 | 1.03E-05 | 0.00349 |
| RPL8 | -1.822 | 1.05E-05 | 0.00349 |
| CLTA | -2.113 | 1.12E-05 | 0.00357 |
| EEF2 | -2.031 | 1.25E-05 | 0.00374 |
| COX4I1 | -2.224 | 1.52E-05 | 0.00428 |
| CLDN3 | -2.605 | 1.54E-05 | 0.00428 |
| FXYD3 | -3.048 | 1.61E-05 | 0.00428 |
| AGR2 | -2.955 | 1.67E-05 | 0.00431 |
| C19orf21 | -2.750 | 2.11E-05 | 0.00530 |
| LGALS3BP | -2.382 | 2.35E-05 | 0.00575 |
| EIF6 | -2.366 | 2.51E-05 | 0.00598 |
| RPL7A | -2.186 | 3.21E-05 | 0.00745 |
| RPL18 | -1.821 | 3.87E-05 | 0.00856 |
| PLAC8 | -3.432 | 4.06E-05 | 0.00863 |
| ITM2C | -2.997 | 4.21E-05 | 0.00863 |
| IFITM1 | -2.362 | 4.35E-05 | 0.00863 |
| ANXA2 | -1.828 | 4.40E-05 | 0.00863 |
| MLPH | -2.816 | 4.46E-05 | 0.00863 |
| SPINK4 | -3.093 | 4.96E-05 | 0.00941 |
| GLTSCR2 | -1.909 | 5.25E-05 | 0.00973 |
| MRPL12 | -2.204 | 5.34E-05 | 0.00973 |
| ATP10B | -2.599 | 5.46E-05 | 0.00976 |
| MUC5B | -2.723 | 6.19E-05 | 0.01049 |
| RPS15 | -1.992 | 6.21E-05 | 0.01049 |
| RPLP0 | -2.528 | 6.57E-05 | 0.01070 |
| PSME1 | -1.827 | 6.77E-05 | 0.01085 |
| EPN1 | -2.629 | 6.94E-05 | 0.01093 |
| CKB | -2.656 | 7.09E-05 | 0.01098 |
| GGT6 | -3.110 | 8.07E-05 | 0.01226 |
| RPLP1 | -2.264 | 8.66E-05 | 0.01249 |
| CYP2S1 | -2.916 | 9.93E-05 | 0.01397 |
| INF2 | -2.215 | 0.000107 | 0.01468 |
| MYH9 | -1.510 | 0.000109 | 0.01468 |
| CBLC | -2.842 | 0.000110 | 0.01468 |
| S100A11 | -2.387 | 0.000112 | 0.01468 |
| GSR | -2.517 | 0.000112 | 0.01468 |
| PABPC1 | -1.836 | 0.000124 | 0.01598 |
| KLF5 | -2.105 | 0.000131 | 0.01667 |
| EPHX2 | -3.140 | 0.000137 | 0.01725 |
| TBRG4 | -2.646 | 0.000150 | 0.01799 |
| AGR3 | -3.020 | 0.000151 | 0.01799 |
| EPS8L3 | -2.308 | 0.000151 | 0.01799 |
| MDH2 | -1.950 | 0.000166 | 0.01922 |
| RNASET2 | -2.342 | 0.000176 | 0.02021 |
| ACOT7 | -2.460 | 0.000178 | 0.02021 |
| EEF1G | -1.405 | 0.000182 | 0.02041 |
| CLTB | -1.721 | 0.000193 | 0.02134 |
| PRKCSH | -1.639 | 0.000203 | 0.02200 |
| ATP5D | -2.714 | 0.000204 | 0.02200 |
| RALY | -1.804 | 0.000210 | 0.02244 |
| CD74 | -2.290 | 0.000219 | 0.02316 |
| CYSTM1 | -2.535 | 0.000242 | 0.02500 |
| ASL | -2.407 | 0.000265 | 0.02673 |
| RPL29 | -2.279 | 0.000268 | 0.02678 |
| HSD11B2 | -2.895 | 0.000271 | 0.02680 |
| UGT2A3 | -3.080 | 0.000285 | 0.02789 |
| TKT | -1.561 | 0.000289 | 0.02793 |
| S100A6 | -1.818 | 0.000346 | 0.03228 |
| DUS1L | -1.667 | 0.000347 | 0.03228 |
| ETFB | -2.540 | 0.000360 | 0.03310 |
| CREG1 | -2.095 | 0.000397 | 0.03545 |
| ALDOA | -1.729 | 0.000404 | 0.03545 |
| TSPAN8 | -2.579 | 0.000409 | 0.03545 |
| MVP | -2.051 | 0.000409 | 0.03545 |
| FTL | -1.913 | 0.000412 | 0.03545 |
| IFI27 | -2.299 | 0.000416 | 0.03545 |
| DAZAP1 | -1.668 | 0.000420 | 0.03545 |
| TFDP1 | -1.929 | 0.000425 | 0.03553 |
| HHLA2 | -2.706 | 0.000463 | 0.03729 |
| PCYT2 | -2.253 | 0.000472 | 0.03751 |
| CISD1 | -2.507 | 0.000489 | 0.03839 |
| TNIP1 | -2.161 | 0.000492 | 0.03839 |
| PITPNM1 | -2.667 | 0.000556 | 0.04197 |
| PRDX2 | -1.966 | 0.000568 | 0.04252 |
| OAF | -2.622 | 0.000576 | 0.04267 |
| RPS19 | -2.047 | 0.000579 | 0.04267 |
| RIN2 | -2.053 | 0.000584 | 0.04271 |
| CHCHD10 | -2.808 | 0.000593 | 0.04287 |
| RPL13A | -2.048 | 0.000600 | 0.04287 |
| AHCYL2 | -2.240 | 0.000608 | 0.04311 |
| PLEKHJ1 | -2.207 | 0.000613 | 0.04313 |
| SDHB | -2.207 | 0.000646 | 0.04477 |
| ABHD11 | -1.972 | 0.000732 | 0.04950 |
| LAD1 | -2.184 | 0.000734 | 0.04950 |
| MIF | -2.197 | 0.000741 | 0.04950 |
| STARD10 | -2.051 | 0.000756 | 0.04980 |
| NR2F6 | -2.771 | 0.000763 | 0.04990 |
