# Supplementary material for: Aberrant DNA Methylation: Implications in Racial Health Disparity
Source: PLoS One. 2016 Apr 25;11(4):e0153125. doi: 10.1371/journal.pone.0153125 (PMC4844165; doi:10.1371/journal.pone.0153125)
Supplement: S5 Table — (DOCX) [file pone.0153125.s006.docx]

**S5 Table. Upregulated genes in AA CRC compared CA CRC, ranked by statistical significance.**

| **Gene** | **Fold Change (log2)** | **p-value** | **FDR** |
| --- | --- | --- | --- |
| THBS2 | 2.192 | 3.22E-07 | 0.00030 |
| MNS1 | 1.920 | 4.73E-07 | 0.00037 |
| BDNF-AS1 | 3.451 | 5.83E-07 | 0.00041 |
| PCA3 | 3.530 | 6.24E-07 | 0.00041 |
| DNM1P46 | 2.911 | 4.53E-06 | 0.00200 |
| CYP1B1 | 3.149 | 1.15E-05 | 0.00357 |
| OBSCN | 2.066 | 1.61E-05 | 0.00428 |
| BCAT1 | 2.980 | 3.63E-05 | 0.00823 |
| RNF224 | 2.672 | 4.27E-05 | 0.00863 |
| ZNF772 | 2.993 | 5.85E-05 | 0.01024 |
| MAP4K4 | 1.452 | 6.52E-05 | 0.01070 |
| EMB | 2.660 | 8.19E-05 | 0.01226 |
| MIR1279 | 1.563 | 8.65E-05 | 0.01249 |
| SLC2A3 | 2.647 | 8.74E-05 | 0.01249 |
| ZFHX4 | 3.021 | 0.00014 | 0.01745 |
| KIF26B | 3.155 | 0.00016 | 0.01922 |
| LOC100505678 | 2.164 | 0.00024 | 0.02483 |
| NGFRAP1 | 1.658 | 0.00025 | 0.02534 |
| IL7 | 1.925 | 0.00033 | 0.03129 |
| LOC441177 | 2.751 | 0.00033 | 0.03158 |
| VCAN | 2.338 | 0.00042 | 0.03545 |
| PCDHA2 | 2.932 | 0.00042 | 0.03545 |
| COL10A1 | 2.565 | 0.00044 | 0.03638 |
| ADMANGPTL3 | 2.281 | 0.00044 | 0.03641 |
| LOC729177 | 2.087 | 0.00045 | 0.03647 |
| IGFBP5 | 1.779 | 0.00047 | 0.03729 |
| CCDC39 | 1.470 | 0.00052 | 0.03997 |
| COL11A1 | 2.394 | 0.00052 | 0.03997 |
| FGF7 | 2.092 | 0.00054 | 0.04096 |
| AMIA3 | 1.307 | 0.00060 | 0.04287 |
| RORA | 1.704 | 0.00063 | 0.04406 |
| POLR2B | 1.872 | 0.00070 | 0.04805 |
| ALPP | 2.864 | 0.00074 | 0.04950 |
| PRRX1 | 2.483 | 0.00075 | 0.04950 |
